# Association between dietary vitamin C and abdominal aortic calcification among the US adults

**DOI:** 10.1186/s12937-023-00889-y

**Published:** 2023-11-15

**Authors:** Jundi Jia, Jie Zhang, Qiao He, Mingqi Wang, Qiyu Liu, Tongxin Wang, Xuanye Chen, Wen Wang, Hao Xu

**Affiliations:** 1 Graduate School, Beijing University of Chinese Medicine, Beijing, China; 2 National Clinical Research Center for Chinese Medicine Cardiology, Xiyuan Hospital, Academy of Chinese Medical Sciences, Beijing, China; 3 National Integrated Traditional and Western Medicine Center for Cardiovascular Disease, China-Japan Friendship Hospital, Beijing, China; 4 Clinical Epidemiology and Evidence-Based Medicine Research Center, West China Hospital, Sichuan University, Chengdu, China; 5 Graduate School, China Academy of Chinese Medical Sciences, Beijing, China

**Keywords:** Vitamin C, Vascular calcification, Abdominal aortic calcification, Cardiovascular disease, NHANES

## Abstract

**Background:**

Cardiovascular disease (CVD) is the leading cause of mortality, and vascular calcification has been highly correlated with CVD events. Abdominal aortic calcification (AAC) has been shown to predict subclinical CVD and incident CVD events. However, the relationship between vitamin C and abdominal aortic calcification remains unclear.

**Objective:**

To investigate the relationship of dietary vitamin C with AAC among the adult population in the US.

**Methods:**

The National Health and Nutrition Examination Survey (NHANES) 2013–2014 provided the data for the cross-sectional study. 2297 subjects (1089 males) were included in the study. Two scoring systems, AAC 24-point scale (Kauppila) and AAC 8-point scale (Schousboe), were used for the measurement of AAC score. Dietary vitamin C intake was calculated as the average of two rounds of 24-h interview recall data and classified in tertiles for analysis. We applied weighted multiple regression analyses to assess the relationship of dietary vitamin C with AAC score and the risk of having AAC. To ensure the robustness of the findings, subgroup and sensitivity analyses were performed. Additionally, smooth curve fittings, using generalized additive models (GAM) were employed to visualize potential nonlinear relationships. Furthermore, an exploratory analysis on the relationship of vitamin C supplements with AAC was also conducted.

**Results:**

The results showed that higher dietary vitamin C intake was related to a reduction in AAC score (AAC-24: β = -0.338, 95% confidence interval [CI] -0.565, -0.111, *P* = 0.004; AAC-8: β = -0.132, 95%CI -0.217, -0.047, *P* = 0.002), and lower risk of AAC (odds ratio [OR] = 0.807, 95%CI 0.659, 0.989, *P* = 0.038). However, the relationship of vitamin C supplements with AAC was not identified.

**Conclusions:**

The study revealed that higher intake of dietary vitamin C rather than vitamin C supplements was related to reduced AAC score and lower risk of AAC, indicating that diets rich in vitamin C are recommended due to its potential benefits for protecting against vascular calcification and CVD among the adult population in the US.

**Supplementary Information:**

The online version contains supplementary material available at 10.1186/s12937-023-00889-y.

## Introduction

Vascular calcification is typified by the pathological deposition of calcium phosphate crystals in the intima of arteries [1], which has been determined to be a risk factor for cardiovascular diseases (CVDs) and is highly linked to the increased risk of atherosclerotic plaque rupture, adverse cardiovascular events and all-cause mortalit [2]. To date, the understanding of the calcification mechanism remains incomplete, and the available treatments for calcification are restrictive. While, animal studies have shown the promising potential of statin [3] and calcium channel blockers (CCBs) in treating vascular calcification, their efficacy in human subjects remains controversial [4–6]. Although sodium thiosulfate has been validated as an effective treatment for vascular calcification in rats [7], further large randomized controlled trials are necessary to confirm its clinical efficacy and safety for human use. Given the association of vascular calcification with CVDs and mortality, and the therapeutic dilemma, the prevention and improvement method of vascular calcification deserve further exploration.

Abdominal aortic calcification (AAC), as a potential indicator related to vascular morbidity and mortality, occurs earlier than coronary artery calcification and has been shown to predict subclinical CVDs and incident CVD events, independently of traditional risk factors [8–10]. Moreover, AAC was found outperforming the Framingham risk score as a sensitive predictive factor of cardiovascular events in a retrospective study [11]. The Multi-Ethnic Study of Atherosclerosis (MESA) study examined the difference in cardiovascular outcomes between AAC and coronary artery calcification (CAC). Both AAC and CAC were identified as independent predictors for coronary heart disease and CVD. Nevertheless, it was AAC, not CAC, that demonstrated a significant association with cardiovascular disease mortality, and AAC was a more robust predictor for all-cause mortality than CAC [12]. Early prevention and treatment of AAC or slowing its progression is essential, even with lesser calcification. A 10-year longitudinal study demonstrated that both a rapid nonlinear increase and a late slow-increasing trajectory of abdominal aortic calcification were associated with an increased risk of death compared with a stable trajectory[adjusted hazard ratios (HR) 1.91; 95% CI 1.02–3.58 and adjusted HR 2.79; 95% CI: 1.44–5.11, respectively] [13]. A 5-year randomized controlled trial of calcium supplementation versus placebo enrolling 1471 healthy women indicated that compared with patients without AAC, the unadjusted HR for the occurrence of CVD events and myocardial infarction in patients with any AAC were 1.7 and 2.3, respectively. The relative risk of myocardial infarction remained significant after 5-year cardiovascular disease risk adjustment based on clinical risk factors. In addition, progression of AAC was significantly associated with incident CVD and incident myocardial infarction, with HR of 1.6 and 2.1, respectively [14]. Therefore, methods of reversing abdominal aortic calcification at any level or methods of slowing the progression of calcification are worth exploring and may be benefit to the patients. The AAC measurement is commonly carried out using the scoring systems of of Kauppila [15] and Schousboe [16].

Dietary approaches have gained significant attention for their role in the primary prevention of CVDs, and other chronic disease [17]. Dietary Vitamin C, widely found in foods including fruits, vegetables, legumes, nuts and whole grains, is an essential dietary requirement for humans, and has been related to cardiovascular health [18, 19]. Vitamin C has antioxidant properties that reduce the production of reactive oxygen species, have anti-inflammatory effects, and the ability to reduce lipid peroxidation [20]. It has been suggested as a possible preventive therapy for CVDs and corresponding mortality [21–23], and may play a part in vascular calcification. A meta-analysis that included three interventional studies and 15 prospective cohort studies demonstrated that higher vitamin C intake was correlated with a lower risk of CVD mortality (RR = 0.79, 95% CI 0.68, 0.89) [24]. Previous observations indicating a lower risk of stroke and coronary heart disease with higher vitamin C intake may be attributed to the protective effect of vitamin C against calcification [25, 26]. Furthermore, vitamin C was discovered to interfere with the process of vascular calcification in a vitro experiment. The study demonstrated that vitamin C significantly reduced calcium accumulation produced and deposited by vascular smooth muscle cells [27]. However, the clinical evidence is lacking.

To our knowledge, there were no clinical studies that reported the relationship of dietary vitamin C with AAC. Thus, we applied cross-sectional research to validate the hypothesis that higher dietary vitamin C intake is in association with reduced AAC score and lower risk of AAC.

## Materials and methods

### Study population

Data were derived from the National Health and Nutrition Examination Survey (NHANES), a cross-sectional investigation aimed at obtaining preliminary information and assessing the health status of the US population. NHANES is conducted by National Center for Health Statistic (NCHS), using a non-institutionalized design that involves a multistage probability sample with stratification. NCHS Ethics Review Board had reviewed the ethics of the NHANES and had approved its conduction. All the subjects signed the informed consent before participating in the survey [28].

**Fig. 1:**
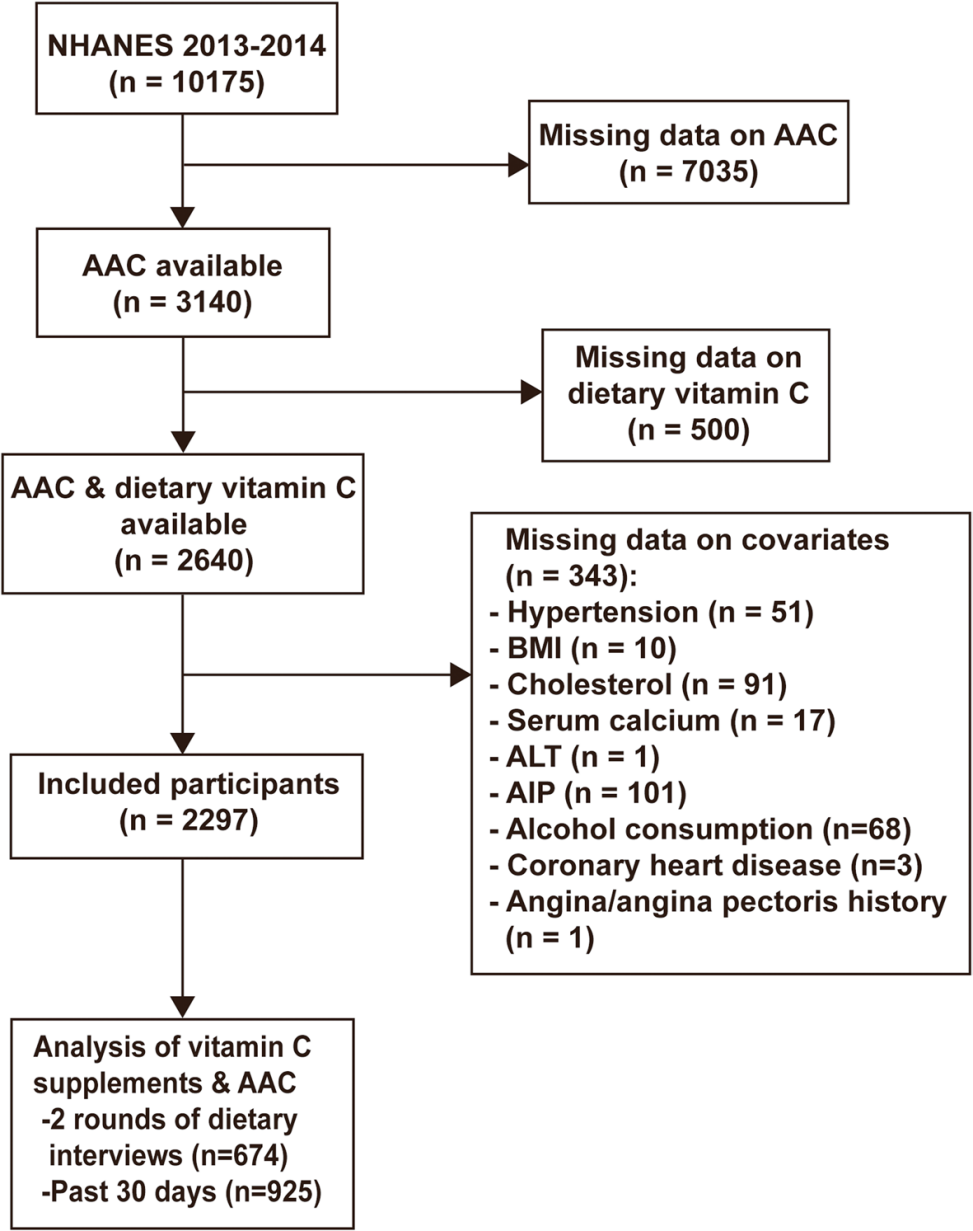
The flowchart

The selection of data from NHANES 2013–2014 cycle was based on its inclusion of AAC information, as no other cycle contained information of vascular calcification. Subjects were limited to those aged ≥ 40 years old, were not pregnant, and did not provide any self-reported radiation exposure over the previous seven-day period, due to the application of dual-energy X-ray absorptiometry (DXA) to measure AAC. We enrolled the subjects with data on both AAC and dietary vitamin C (*n* = 2640). Finally, the study included 2297 subjects after removing those with missing data on covariates. To further explore the relationship of vitamin C supplements with AAC, we also extracted the information of participants with data on vitamin C supplements (Fig. 1).

### Exposure and outcome definitions

Dietary vitamin C as a continuous variable was the primary exposure in this study. While it was also classified into tertiles as a categorical variable for analysis, serving as the secondary exposure. Dietary data of NHANES, including information on food components, nutrients, and energy, was charged by Food Surveys Research Group (FSRG) regarding agriculture in the US. Dietary vitamin C was calculated as the average of two rounds of 24-h interview recall data. The initially collected recall was obtained in-person at the Mobile Examination Center (MEC), while the second interview was carried out over the phone after a period of 3 to 10 days.

AAC was the dependent variable in the study. The examination section of NHANES provided the data of AAC. The AAC was presented by images of lateral spine obtained through DXA, which could reduce radiant exposure. The lateral spine scans provide image resolution comparable to that observed in standard X-rays.The technologists were certified and annually trained. To determine the AAC score, Kauppila's approach (AAC-24 score) was employed, which divided the aorta's anterior and posterior walls into four sections adjacent to the lumbar vertebrae L1-L4. Each segment of the aorta was given a score ranging from 0 to 6 points based on the extent of calcification observed, resulting in a total score range of 0 to 24. AAC was deemed present if the Kauppila score was greater than 0, as evidenced by prior study [29]. All patients with AAC score were included in the analysis, not only for patients with severe calcification, to better exploring whether the dietary vitamin C could prevent or reverse the presence of calcification in the general population. In addition, we applied the Schousboe score (AAC-8 score) ranged from 0 to 8, as the outcome variable for sensitivity analysis. AAC-8 scoring method is similar to AAC-24 scoring, having the advantage of being less susceptible to the influence of small calcification distributed across 8 segments. However, AAC-8 measurement requires more expertise and skill [30].

### Covariates

According to the clinical significance, demographics, economic status, metabolic indicators, comorbidities, and lifestyles were considered as confounding factors. The following covariates were included in our study: age, gender, body mass index (BMI), race/ethnicity, poverty ratio, cholesterol, creatinine, serum calcium, serum phosphorus, atherogenic index of plasma (AIP), aspartate aminotransferase (AST), alanine aminotransferase (ALT), diabetes, hypertension, angina/angina pectoris history, coronary heart disease history, alcohol consumption, smoking status, and daily energy intake. The presentation of covariates was shown in the Supplementary Table 1. The measurements for the covariates mentioned above are accessible on the site: www.cdc.gov/nchs/nhanes/.

### Sensitivity, subgroup and exploratory analyses

To ensure the robustness of the results, AAC-8 scoring method for the vascular calcification was used for sensitivity analysis. Subgroup analyses stratified by gender, BMI (BMI ≥ 30 kg/m^2^; BMI < 30 kg/m^2^) [31], age (age ≥ 65 years; age < 65 years) [32], diabetes, hypertension, angina/angina pectoris, coronary heart disease, alcohol consumption and smoking status were conducted to access the stability of the results among different population stratification. Moreover, vitamin C supplement was used as an exploratory exposure in the study. We applied exploratory analysis on the relationship between vitamin C supplements and AAC to investigate the effect of different sources of the vitamin C on vascular calcification. Vitamin C supplements were determined by averaging the data from two rounds of 24-h recall interviews. Besides, data on vitamin C supplements within the past 30 days provided directly by NHANES in the dietary data module were also included in the analysis.

### Statistical analyses

Due to the complexity of the NHANES design, which includes multiple stages of cluster, weighted analysis was conducted according to the recommendation of the NCHS. Weighted means with standard error (SE) were used to describe continuous variables, whereas, frequency (percentage) was used to describe categorical variables. Dietary vitamins C was divided into tertiles for analysis. The difference in variables among different groups was determined using a weighted chi-squared test for categorical variables and a weighted linear regression model for continuous variables. We performed weighted multiple regression analyses to investigate the independent relationship of dietary vitamin C and vitamin C supplements with AAC after adjusting for covariates. Weighted logistic regression model was used for analyzing the association of vitamin C with AAC risk. Using stratified multivariate regression, we performed subgroup analysis, and all covariates except the stratification variable itself were adjusted. P for interaction was based on the log likelihood ratio test to assess the heterogeneity of relationship between the subgroups. Furthermore, we employed GAM-based smooth curve fittings to examine whether the relationship of dietary vitamin C with AAC was nonlinear. If the value of two-sided P was less than 0.05, it was considered statistically significant. We performed statistical analysis using R version 3.4.3 (http://www.R-project.org, The R Foundation) and Empower software (www.empowerstats.com; X&Y solutions, Inc., Boston MA).

## Results

### Baseline

The weighted characteristics of subjects are presented in Table 1. 2297 subjects with mean age of 57.81 ± 11.41 years were included in this study, of whom 51.34% were female and 71.72% were non-Hispanic White. The average dietary vitamin C intake was (82.46 ± 70.09) mg/day, and the mean intake of dietary vitamin C for tertiles was (21.57 ± 10.78) mg/day, (64.44 ± 15.47) mg/day, and (160.90 ± 64.85) mg/day, respectively. Subjects who had higher dietary intake of vitamin C tended to be older and have higher daily energy intake, and were less likely to have diabetes, angina/angina pectoris history and belong to a lower socioeconomic status. Furthermore, they were more likely to have lower BMI, cholesterol levels, serum phosphorus levels and AIP.

**Table 1:** Characteristic of participants in the study, weighted

	Tertiles of dietary vitamin C intake (mg/day)				
	Tertile1: 21.57 ± 10.78 (*n* = 766)	Tertile 2: 64.44 ± 15.47 (*n* = 765)	Tertile 3: 160.90 ± 64.85 (*n* = 766)	All: 82.46 ± 70.09 (*n* = 2297)	*P*-value
**Age (years)**	56.82 ± 11.15	57.16 ± 11.28	59.43 ± 11.60	57.81 ± 11.41	< 0.0001
**Gender, N (%)**					
Male	352 (50.88)	357 (45.57)	380 (49.56)	1089 (48.66)	
Female	414 (49.12)	408 (54.43)	386 (50.44)	1208 (51.34)	0.0965
**Race/ethnicity, N (%)**					
Hispanic	133 (9.25)	190 (12.34)	189 (13.39)	512 (11.67)	
Non-Hispanic White	411 (74.18)	352 (71.26)	330 (69.76)	1093 (71.72)	
Non-Hispanic Black	158 (10.55)	131 (8.35)	141 (9.31)	430 (9.40)	
Others	64 (6.02)	92 (8.05)	106 (7.54)	262 (7.21)	0.0751
**Poverty ratio, N (%)**					
≥ 1.3	508 (79.18)	573 (84.97)	585 (84.26)	1666 (82.81)	
< 1.3	258 (20.82)	192 (15.03)	181 (15.74)	631 (17.19)	0.0063
**BMI(kg/m**^**2**^**)**	28.99 ± 5.26	28.70 ± 5.44	27.88 ± 5.77	28.52 ± 5.52	0.0003
**Cholesterol(mg/dL)**	201.25 ± 42.66	199.06 ± 42.08	193.69 ± 38.49	197.99 ± 41.24	0.0011
**Creatinine(mg/dL)**	0.94 ± 0.46	0.91 ± 0.30	0.93 ± 0.27	0.93 ± 0.35	0.1168
**Serum phosphorus (mg/dL)**	3.80 ± 0.56	3.88 ± 0.57	3.80 ± 0.55	3.82 ± 0.56	0.0078
**Serum calcium (mg/dL)**	9.43 ± 0.34	9.48 ± 0.37	9.47 ± 0.38	9.46 ± 0.37	0.0434
**ALT**	24.77 ± 15.44	24.68 ± 12.61	23.99 ± 23.35	24.48 ± 17.73	0.6413
**AST**	25.79 ± 16.53	24.54 ± 9.11	25.25 ± 14.93	25.19 ± 13.89	0.2149
**AIP**	3.36 ± 2.44	3.06 ± 2.65	2.93 ± 2.45	3.12 ± 2.52	0.0030
**Hypertension, N (%)**					
Yes	265 (31.14)	252 (27.21)	272 (31.91)	789 (30.08)	
No	501 (68.86)	513 (72.79)	494 (68.09)	1508 (69.92)	0.0977
**Diabetes, N (%)**					
Yes	184 (17.68)	185 (17.72)	135 (12.94)	504 (16.11)	
No	582 (82.32)	580 (82.28)	631 (87.06)	1793 (83.89)	0.0136
**Coronary heart disease history, N (%)**					
Yes	48 (7.24)	36 (4.12)	48 (5.34)	132 (5.56)	
No	718 (92.76)	729 (95.88)	718 (94.66)	2165 (94.44)	0.0269
**Angina/angina pectoris history, N (%)**					
Yes	37 (4.64)	23 (2.93)	20 (2.44)	80 (3.33)	
No	729 (95.36)	742 (97.07)	746 (97.56)	2217 (96.67)	0.0428
**Smoking status, N (%)**					
Yes	596 (74.37)	552 (69.13)	548 (73.65)	1696 (72.40)	
No	170 (25.63)	213 (30.87)	218 (26.35)	601 (27.60)	0.1491
**Alcohol consumption, N (%)**					
Yes	664 (89.27)	659 (89.00)	645 (87.74)	1968 (88.67)	
No	102 (10.73)	106 (11.00)	121 (12.26)	329 (11.33)	0.6026
**Daily energy intake (kcal/d)**	1869.75 ± 700.64	2034.95 ± 812.14	2140.86 ± 712.22	2015.64 ± 751.87	< 0.0001

Percentage for Categorical variables, mean ± SE for continuous variables. *BMI* Body mass index, *AAC* Abdominal aortic calcification, *ALT* Alanine aminotransferase, *AST* Aspartate aminotransferase, *AIP* Atherogenic index of plasma

### Inverse association of dietary vitamin C intake with AAC score and the risk of AAC

We conducted three weighted multiple regression models to evaluate the association of dietary vitamin C intake with AAC (Table 2). Age, race, gender, and BMI were adjusted in Model 1. Metabolic factors (cholesterol, creatinine, serum phosphorus, serum calcium, ALT, AST, and AIP) were added to adjust in Model 2. Model 3 was a fully adjusted model with the addition of economic status, comorbidities, and lifestyle factors (poverty ratio, hypertension, diabetes, angina/angina pectoris history, coronary heart disease history, smoking status, alcohol consumption, and daily energy intake). The inverse relationship of dietary vitamin C with AAC-24 score was evident in all models (Model 1: β = -0.376, 95%CI -0.550, -0.203, *P* < 0.001; Model 2: β = -0.368, 95%CI -0.541, -0.195, *P* < 0.001; Model 3: β = -0.338, 95%CI -0.565, -0.111, *P* = 0.004). It could be understood that each 100 mg increment in dietary vitamin C intake might be related to 0.338 decreases in AAC-24 score in the fully adjusted model. Furthermore, high-tertile group was related to decreased AAC-24 score (Model 3, T2: β = -0.484, 95%CI -0.870, -0.098, *P* = 0.014, T3: β = -0.723, 95%CI -1.128, -0.319, *P* < 0.001, *P* for trend < 0.001). When AAC score was treated as a categorical variable, inverse relationship was observed between dietary vitamin C intake and the risk of AAC (Model 3: OR = 0.807, 95%CI 0.659, 0.989, *P* = 0.038), which indicated that each 100 mg increment in dietary vitamin C intake might be related to 19.3% reduced risk of AAC.

**Table 2:** Associations of dietary vitamin C intake with AAC score and the risk of AAC

**Dietary vitamin C (10**^**−2**^** mg/day)**	Model 1		Model 2		Model 3	
	β /OR (95%CI)	*P* value	β /OR (95%CI)	*P* value	β /OR (95%CI)	*P* value
**AAC risk**	0.815 (0.708, 0.939)	0.005	0.816 (0.707, 0.941)	0.005	0.807 (0.659, 0.989)	0.038
**AAC-24 score**	-0.376 (-0.550, -0.203)	< 0.001	-0.368 (-0.541, -0.195)	< 0.001	-0.338 (-0.565, -0.111)	0.004
Groups of vitamin C intake						
T1	Reference		Reference		Reference	
T2	-0.283 (-0.580, 0.015)	0.0625	-0.246 (-0.543, 0.051)	0.104	-0.484 (-0.870, -0.098)	0.014
T3	-0.523 (-0.823, -0.223)	< 0.001	-0.518 (-0.818, -0.219)	< 0.001	-0.723 (-1.128, -0.319)	< 0.001
P for trend	< 0.001		< 0.001		< 0.001	
**AAC-8 score**	-0.146 (-0.211, -0.082)	< 0.001	-0.144 (-0.209, -0.080)	< 0.001	-0.132 (-0.217, -0.047)	0.002
Groups of vitamin C intake						
T1	Reference		Reference		Reference	
T2	-0.062 (-0.172, 0.050)	0.276	-0.047 (-0.158, 0.063)	0.404	-0.134 (-0.279, 0.011)	0.070
T3	-0.192 (-0.303, -0.080)	< 0.001	-0.191 (-0.303, -0.080)	< 0.001	-0.259 (-0.411, -0.107)	< 0.001
P for trend	< 0.001		< 0.001		< 0.001	

Model 1: age, gender, race and BMI were adjusted; Model 2: Model 1 + cholesterol, creatinine, serum phosphorus serum calcium, ALT, AST and AIP; Model 3: Model2 + poverty ratio, hypertension, diabetes, coronary heart disease, angina/angina pectoris history, smoking status, alcohol consumption and daily energy intake

*Abbreviation*: *CI* Confidence intervals, *OR* Odds ratio

**Fig. 2:**
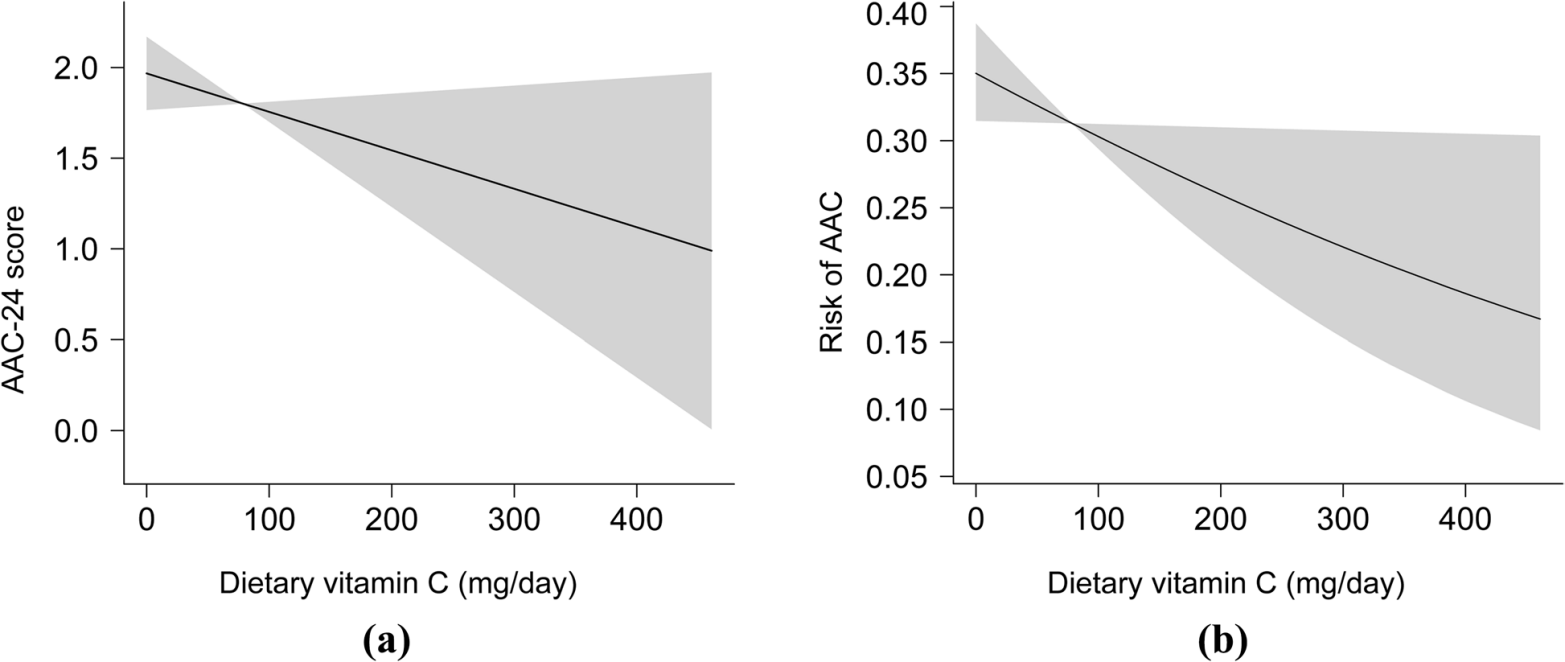
The association of dietary vitamin C with AAC score and the risk of AAC based on generalized additive models. The shaded areas represent 95% confidence intervals. **a** The linear relationship between HEI-2015 and AAC-24 score. **b** The linear relationship between HEI-2015 and the risk of AAC

To explore the potential non-linear association between dietary vitamin C intake and AAC, smooth curve fittings based on GAM were conducted. After the adjustment of all covariates, the results indicated that a linear relationship existed between dietary vitamin C intake and AAC (Fig. 2). The trends were in line with the regression analyses.

We also observed the inverse relationship of dietary vitamin C intake with AAC-8 score in all models, and subjects in the second and third tertiles had lower AAC-8 score than those in the first tertile, which indicated that the results were stable (Table 2).

### Consistent results in different stratification of subgroups

We conducted the subgroup analysis, grouped by gender, age, BMI, hypertension, diabetes, angina/angina pectoris history, coronary heart disease history, alcohol consumption and smoking status, to explore whether the inverse relationship of dietary vitamin C intake and AAC is stable among the different population (Table 3). After adjusting for covariates, the trends of β-coefficients remained steady across all stratification, implying that the relationship of dietary vitamin C intake with AAC score was robust, with no statistically significant interaction found.

**Table 3:** Subgroup analysis

**Stratification**	**N(%)**	AAC-24 score		AAC-8 score	
		β (95%CI)	*P* for interaction	β (95%CI)	*P* for interaction
**Gender (%)**					
Male	1089 (48.66)	-0.506 (-0.812, -0.201)	0.104	-0.202 (-0.317, -0.087)	0.073
Female	1208 (51.34)	-0.141 (-0.471, 0.189)		-0.051 (-0.174, 0.073)	
**Age (years)**					
< 65	1520 (66.17)	-0.205 (-0.469, 0.060)	0.607	-0.077 (-0.176, 0.022)	0.468
≥ 65	777 (33.83)	-0.337 (-0.776, 0.102)		-0.147 (-0.312, 0.018)	
**BMI (kg/m**^**2**^**)**					
< 30	1462 (63.65)	-0.257 (-0.550, 0.037)	0.250	-0.094 (-0.204, 0.016)	0.169
≥ 30	835 (36.35)	-0.519 (-0.869, -0.170)		-0.212 (-0.343, -0.080)	
**Hypertension (%)**					
Yes	789 (30.08)	-0.519 (-0.897, -0.142)	0.234	-0.214 (-0.356, -0.072)	0.153
No	1508 (69.92)	-0.240 (-0.519, 0.039)		-0.088 (-0.193, 0.016)	
**Diabetes (%)**					
Yes	504 (16.11)	-0.717 (-1.327, -0.108)	0.184	-0.301 (-0.529, -0.073)	0.115
No	1793 (83.89)	-0.280 (-0.523, -0.037)		-0.106 (-0.197, -0.016)	
**Coronary heart disease (%)**					
Yes	132 (5.56)	-0.560 (-1.429, 0.308)	0.599	-0.233 (-0.558, 0.093)	0.527
No	2165 (94.44)	-0.324 (-0.557, -0.090)		-0.126 (-0.213, -0.038)	
**Angina/angina pectoris (%)**					
Yes	80 (3.33)	-0.358 (-1.330, 0.615)	0.968	-0.211 (-0.574, 0.152)	0.661
No	2217 (96.67)	-0.338 (-0.573, -0.103)		-0.128 (-0.216, -0.041)	
**Smoking status (%)**					
Yes	1696 (72.40)	-0.345 (-0.619, -0.071)	0.935	-0.135 (-0.237, -0.033)	0.926
No	601 (27.60)	-0.325 (-0.729, 0.079)		-0.127 (-0.277, 0.024)	
**Alcohol consumption (%)**					
Yes	1968 (88.67)	-0.339 (-0.579, -0.099)	0.998	-0.135 (-0.224, -0.045)	0.880
No	329 (11.33)	-0.338 (-1.073, 0.398)		-0.113 (-0.387, 0.162)	

The subgroup analyses were adjusted for all covariates except the stratification variable itself

### No inverse association of vitamin C supplements with AAC

To explore the effect of vitamin C supplements on vascular calcification, we conducted multiple regression analyses between vitamin C supplements and AAC (Supplementary Table 2). The results showed that neither two rounds of dietary interviews nor the past 30 days of vitamin C supplements intake were related to AAC score and the risk of having AAC.

## Discussion

The research investigated the relationship of dietary vitamin C intake with AAC, by applying NHANES 2013–2014 data that is both extensive and representative. The findings suggested that increased dietary vitamin C intake was related to reduced AAC score and lower risk of AAC, and this association was observed across different population stratification with steady consistency. Moreover, there was no inverse relationship of vitamin C supplements with AAC, implying that not all sources of vitamin C are beneficial for vascular calcification and indicating the importance of dietary sources of vitamin C.

### Vascular calcification with oxidative stress and inflammation

Vascular calcification happens in the intimal and middle layers of the arteries. It has been considered an active osteogenic process of vascular cells, mainly vascular smooth muscle cells (VSMCs), similar to the formation of osteoblasts [33]. The mechanisms contributing to VSMCs differentiation have not been fully elucidated, although the major drivers identified so far are oxidative stress, inflammation, aging, and uremia [34]. Exposure of VSMCs to hydrogen peroxide or oxidized low-density lipoprotein implying the accumulation of ROS leads to the increased expression of the osteogenic transcription factor Runx2 [35, 36], which might drive the osteocytic VSMC phenotype and trigger vascular calcification. An animal experiment demonstrated that the antioxidant Tempol reduced aortic and systemic oxidative stress levels, and inhibited osteogenic differentiation of VSMCs and arterial calcification in uremic rats [37].

Furthermore, inflammation was discovered to be linked to osteogenic activity and vascular calcification in the cardiovascular system by distinct near-infrared fluorescent nanoparticle probes [38]. Inflammatory cells, especially macrophages/monocyte, were considered to regulate the phenotypic differentiation of VSMCs [39, 40]. The secretion of pro-inflammatory factors by activated macrophages stimulates the differentiation of VSMCs into bone cells [41]. TNF-α was identified as a pro-inflammatory factor that enhances the expression of alkaline phosphatase (ALP) through the NF-κB pathway, which then facilitates calcification in VSMCs [42]. Additionally, TNF-α is found to take a significant part in the process of medial calcification in people with diabetes and CKD [43]. Interleukin-1β (IL-1β), produced mainly by Rac1 of macrophages, can also promote calcification and is used as a predictive factor of cardiovascular mortality in patients with high coronary calcium burden[40]. Besides, IL-6 and Oncostatin M (OSM) could also realize the differentiation of VSMCs to osteoblast [44, 45].

### The antioxidant and anti-inflammatory effects of vitamin C

Vitamin C, named as ascorbic acid, is a water-soluble natural ingredient found in food and could also be used as a dietary supplement. Due to its antioxidant, collagen-stabilizing, immune modulatory actions, and inflammation-slowing effects [1, 46, 47], there is growing interest in exploring the relationship between vitamin C and chronic diseases involving the above mechanisms.

Moreover, experiments in vitro showed that vitamin C not only decrease the generation of superoxide radicals, hydrogen peroxide, and other reactive oxygen species (ROS) by inhibiting the Jak2/Stat1/IRF1 signaling pathway in endothelial cells [48], and it could also inhibit NF-κB-mediated inflammatory reaction [49] and suppress the synthesis of pro-inflammatory cytokine IL-6 and TNF-α in monocytes [50]. Hence, dietary vitamin C is likely to have a therapeutic effect on vascular calcification through antioxidant and anti-inflammatory actions. In a cross-sectional study of 7607 cases from the NHANES 2003–2006 surveys, subjects with high dietary vitamin C intake were related to lower levels of inflammatory biomarkers [51]. A randomized controlled trial indicated that vitamin C was beneficial in improving oxidative stress, inflammation, and endothelial dysfunction induced by acute hypoglycemia in patients with type 1 diabetes [52].

### The potential benefit of vitamin C for vascular calcification

Previous studies have investigated the association between dietary factors and vascular calcification, and it has garnered significant attention from researchers. Vitamin K and vitamin E were found to attenuate the development of vascular calcification in animal experiments [53, 54]. A cross-sectional survey of 2535 participants indicated that higher dietary zinc intake was related to decreased risk of severe AAC among US adults [55]. Vitamin C has possible beneficial effects on cardiovascular health. A meta-analysis including 69 prospective studies revealed that greater dietary vitamin C intake was correlated with decreased risk of cardiovascular disease, total cancer, and all-cause mortality [18]. However, its connection with vascular calcification remains unclear. A cellular experiment demonstrated that vitamin C reduced calcification accumulation produced by VSMCs in the optimum extracellular matrix. This process was accompanied by reduced alkaline phosphatase activity and the expression of osteoblast marker protein, implying that the osteogenic transformation of VSMCs was blocked [27]. Dietary vitamin C may inhibit the differentiation of VSMCs and prevent vascular calcification through anti-inflammatory and antioxidant effects. Further research is still required to clarify the association and mechanisms.

Our results indicated that vitamin C supplements were not beneficial for vascular calcification, which were consistent with the results of previous randomized controlled studies and mendelian randomization studies [18, 56–58] on vitamin C supplements and CVDs, implying that fruits and vegetables rich in high amounts of natural vitamin C were recommended instead of dietary supplements. The possible reason for this result is that when vitamin C is extracted from a natural product, the intermediate product of this extraction or the process of artificial synthesis may lead to reduced bioavailability of the vitamin in plasma and decreased effects [59]. It was shown that vitamin C from fruit (kiwifruit) resulted in higher levels of ascorbate and superior bioavailability compared to synthetic vitamin C in mice with vitamin C deficienc [60].

### Strengths and limitations

Of note, some strengths could be found in the study. It might be the first research to investigate the relationship of dietary vitamin C with AAC and may provide some support for future studies. Concerning the sensitivity and subgroup analyses, the findings consistently supported the relationship of dietary vitamin C intake with AAC, demonstrating the robustness of the conclusion. Moreover, an exploratory analysis of the relationship of vitamin C supplements with AAC was performed to investigate the effect of different sources of vitamin C on vascular calcification, suggesting that dietary sources of vitamin C are beneficial for vascular calcification rather than supplements. Finally, as a result of utilizing a sample that is representative of the population, the findings can be generally applied to the adult population in the United States.

The study also had certain limitations. First, as the research was conducted in a cross-sectional manner, it is not probable to infer a causal relationship of dietary vitamin C with AAC. Therefore, further longitudinal studies are necessary to elucidate the findings. Second, dietary data were collected via 24-h interview recall, which had the potential for recall bias. Third, NHANES 2013–2014 lacks data on serum vitamin C. Therefore, the relationship of serum vitamins C with AAC cannot be further explored. Additionally, it’s unavoidable that uncollected or unknown confounding factors may influence the study results. For instance, the use of medication may have an effect on the absorption of vitamin C or on vascular calcification. Finally, NHANES collects data on the American population. Due to the constraints of AAC measurement, the study only included individuals who were aged 40 years or above. Our study was centered towards the individuals aged 40 years or older within the general adult population, as this demographic is at a higher risk of developing vascular calcification. Focusing on this group is important in the prevention of calcification of blood vessels. Nevertheless, whether the findings are applied to the populations of different countries and other age groups needs further exploration.

## Conclusion

The study conducted in a cross-sectional design indicated that there is an inverse relationship of dietary intake of vitamin C with the risk of AAC and AAC score. The findings suggest that increased vitamin C intake through diet could have a positive effect on reducing vascular calcification in adults, providing a new perspective on potential dietary approaches to prevent vascular calcification and CVDs. Although more prospective and mechanistic studies are needed to provide additional evidence and support this conclusion in the future.

### Supplementary Information

**Additional file 1: Supplementary Table 1. **Description of covariates. **Supplementary Table 2.** Associations of vitamin C supplements with AAC score and the risk of AAC.

## Data Availability

The data used in the study were obtained from NHANES: https://www.cdc.gov/nchs/nhanes/.
