# Supplementary material for: Association between dietary vitamin C and abdominal aortic calcification among the US adults
Source: Nutr J. 2023 Nov 15;22:58. doi: 10.1186/s12937-023-00889-y (PMC10647183; doi:10.1186/s12937-023-00889-y)
Supplement: Supplementary file 1 — Additional file 1: Supplementary Table 1. Description of covariates. Supplementary Table 2. Associations of vitamin C supplements with AAC score and the risk of AAC. [file 12937_2023_889_MOESM1_ESM.docx]

Supplementary Material

***Association between dietary vitamin C and abdominal aortic calcification among the US Adults***

Jundi Jia, Jie Zhang, Qiao He, Mingqi Wang, Qiyu Liu, Tongxin Wang, Xuanye Chen, Wen Wang^*^ and Hao Xu^*^

**Supplementary Table 1.** Description of covariates.

**Supplementary Table 2.** Associations of vitamin C supplements with AAC score and the risk of AAC.

| **Variable** | **Section** | **Description** |
| --- | --- | --- |
| age | Demographics Data | Age in years of the participant at the time of screening. |
| gender | Demographics Data | Gender of the participant. |
| race/ethnicity | Demographics Data |  |
| poverty ratio | Demographics Data | The poverty ratio was calculated by dividing family income by the 2013 to 2014 Department of Health and Human Services (HHS) poverty guidelines, specific to family size, as well as the appropriate year and state. |
| body mass index (BMI) | Examination Data | kg/m**2. |
| cholesterol | Laboratory Data | - |
| creatinine | Laboratory Data | - |
| serum phosphorus | Laboratory Data | - |
| serum calcium | Laboratory Data | - |
| Alanine aminotransferase(ALT) | Laboratory Data | - |
| Aspartate aminotransferase(AST ) | Laboratory Data | - |
| Atherogenic index of plasma(AIP) | Laboratory Data | log(triglycerides/high-density lipoprotein cholesterol[HDL-C]-cholesterol). |
| diabetes | Questionnaire Data | The next questions are about specific medical conditions. {Other than during pregnancy, {have you/has SP}/ {Have you/Has SP}} ever been told by a doctor or health professional that {you have/{he/she/SP} has} diabetes or sugar diabetes? |
|  | Laboratory Data | Glycohemoglobin> 6.5% |
|  | Laboratory Data | Fasting glucose≥7.0 mmol/L |
|  | Laboratory Data | random blood glucose≥11.1 mmol/L |
|  | Laboratory Data | Two Hour Glucose (OGTT)≥11.1 mmol/L |
| hypertension | Questionnaire Data | {Have you/Has SP} ever been told by a doctor or other health professional that {you/s/he} had hypertension, also called high blood pressure? |
|  | Examination Data | Average SBP≥140 mmHg, average DBP≥90 mmHg |
|  |  |  |
| coronary heart disease history | Questionnaire Data | Has a doctor or other health professional ever told {you/SP} that {you/s/he} . . .had coronary (kor-o-nare-ee) heart disease? |
| angina/angina pectoris history | Questionnaire Data | Has a doctor or other health professional ever told {you/SP} that {you/s/he} . . .had angina (an-gi-na), also called angina pectoris? |
| smoking status | Questionnaire Data | Smoking is defined as “smoked at least 100 cigarettes in life”. |
| alcohol consumption | Questionnaire Data | In {your/SP's} entire life, {have you/has he/ has she} had at least 12 drinks of any type of alcoholic beverage? By a drink, I mean a 12 oz. beer, a 5 oz. glass of wine, or one and half ounces of liquor. |
| daily energy intake | Dietary Data | the average of two rounds of 24-hour interview recall data on energy intake. |

**Supplementary Table 1.** Description of covariates.

| Vitamin C supplements (10^-2^mg/day) | Model 1 | | Model 2 | | Model 3 | |
| --- | --- | --- | --- | --- | --- | --- |
|  | β/OR (95%CI) | *P* value | β/OR (95%CI) | *P* value | β/OR (95%CI) | *P* value |
| **Vitamin C supplements**  **(2 rounds of dietary interviews)** |  |  |  |  |  |  |
| AAC-24 score | 0.013 (-0.048, 0.050) | 0.960 | 0.008 (-0.041, 0.057) | 0.748 | 0.008 (-0.043, 0.060) | 0.746 |
| AAC-8 score | -0.002 (-0.019, 0.016) | 0.862 | 0.001 (-0.017, 0.019) | 0.911 | 0.001 (-0.019, 0.019) | 0.967 |
| AAC risk | 0.980 (0.938, 1.023) | 0.355 | 0.986 (0.944, 1.031) | 0.536 | 0.965 (0.892, 1.044) | 0.373 |
| **Vitamin C supplements (Past 30 days)** |  |  |  |  |  |  |
| AAC-24 score | 0.017 (-0.032, 0.066) | 0.499 | 0.020 (-0.029, 0.069) | 0.432 | 0.001 (-0.061, 0.063) | 0.966 |
| AAC-8 score | 0.002 (-0.017, 0.020) | 0.879 | 0.002 (-0.016, 0.020) | 0.810 | -0.004 (-0.028, 0.019) | 0.718 |
| AAC risk | 0.985 (0.949, 1.023) | 0.438 | 0.989 (0.953, 1.027) | 0.565 | 0.975 (0.926, 1.026) | 0.330 |

**Supplementary Table 2.** Associations of vitamin C supplements with AAC score and the risk of AAC. Model 1: age, gender, race and BMI were adjusted; Model 2: Model 1 + cholesterol, creatinine, serum phosphorus serum calcium, ALT, AST and AIP; Model 3: Model2 + poverty ratio, hypertension, diabetes, coronary heart disease, angina/angina pectoris history, smoking status, alcohol consumption and daily energy intake. Abbreviation: CI, confidence intervals.OR, odds ratio.
